# Supplementary material for: Bioinspired handheld time-share driven robot with expandable DoFs
Source: Nat Commun. 2024 Jan 26;15:768. doi: 10.1038/s41467-024-44993-x (PMC10817928; doi:10.1038/s41467-024-44993-x)
Supplement: Supplementary file 1 — Supplementary Information [file 41467_2024_44993_MOESM1_ESM.pdf]

Supplementary Information for

Bioinspired handheld time-share driven robot with expandable DoFs

Yunjiang Wang,[1] Xinben Hu,[2,3] Luhang Cui,[1] Xuan Xiao,[1]
Keji Yang,[1] Yongjian Zhu,[2,3]* Haoran Jin[1]*

Corresponding authors: Haoran Jin (jinhr@zju.edu.cn); Yongjian Zhu (neurosurgery@zju.edu.cn).

Affiliations:
1Key Laboratory of Fluid Power and Mechatronic Systems, Department of Mechanical Engineering, Zhejiang University, Hangzhou 310058, China.
2Department of Neurosurgery, Second Affiliated Hospital of Zhejiang University School of Medicine, Hangzhou 310009, China.
3Key Laboratory of Precise Treatment and Clinical Translational Research of Neurological Diseases, Hangzhou, 310005, China.

The PDF file includes:

Supplementary Methods

Supplementary Figure 1. Details of the stainless-steel braided hose.

Supplementary Figure 2. A schematic view of the planar steerable tube.

Supplementary Figure 3. The manufacturing process of the flexible inner tube.

Supplementary Figure 4. The fabrication process of one bending module.

Supplementary Figure 5. A schematic view of the time-share driven robot.

Supplementary Figure 6. Modeling of one bending module.

Supplementary Figure 7. Modeling of the time-share driven robot.

Supplementary Figure 8. Demonstration of diagnosis experiment in human stomach model.

Supplementary Figure 9. Demonstration of therapy experiment in ex vivo porcine stomach.

Supplementary Table 1. Comparison of this work with continuum robot designs.

Supplementary Table 2. Parameters of the stainless-steel braided hoses.

Supplementary Table 3. Parameters of the rigid resin and 3D-print process.

Other Supplementary Material for this manuscript includes the following:

Supplementary Movies 1 to 10

Supplementary Methods

1. Comparison of this work with continuum robot designs.

The comparison of main continuum robot designs is listed in Supplementary Table 1. Limited by the manufacturing process, it is prohibitive to build more than two segments in tendon-driven and multi-backbone designs, and more than three segments in concentric tube design. Good maneuverability for their designs, though, the DoFs are limited to six to keep a satisfying footprint for surgery application. The proposed robot is actuated by a flexible rotation shaft that adapts and passes through a tortuous path with a minimal footprint. Several bending modules are actuated by the rotation of the shaft based on the time-share driven mechanism. Benefiting from this mechanism, it is feasible to expand the number of segments, while the cross-section of the manipulator remains the same.

Supplementary Table 1. Comparison of this work with continuum robot designs

Continuum Designs for surgery	Controllable DoFs (Potential to be more than six)	Light-weight (Hand-held)	Task-oriented reconfiguration in situ	Model and control
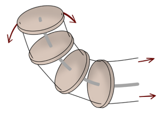 Tendon driven (*28~31*)	★★★☆☆ (1~2) $DoFs/\mathrm{seg}$ × (1~3) seg	★★★☆☆ For manual control instruments	★☆☆☆☆ Tendons cannot be settled in situ	★★★☆☆ Close to constant curvature
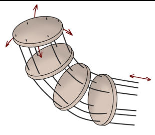 Multi-backbone (*23,27*)	★★★☆☆ (2~3) $DoFs/\mathrm{seg}$ × (1~2) seg	★☆☆☆☆ Large and heavy motor package	★☆☆☆☆ Tendons cannot be settled in situ	★★★★☆ Fit well with constant curvature
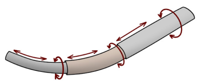 Concentric tube (*12*)	★★★☆☆ 2 $DoFs/\mathrm{seg}$ × (2~3) seg	★★★★☆ Simple transmission structure	★★★☆☆ Simple assembly	★★☆☆☆ complicated by torsional deformation
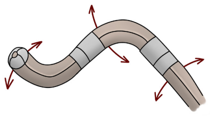 Soft robot (*11*)	★★★★★ (1~3) $DoFs/\mathrm{seg}$ × $n\ seg$	★☆☆☆☆ Air/water pumps are heavy and noisy	★★★★☆ Modular assembled	★☆☆☆☆ complex connection between actuation and robot deformation
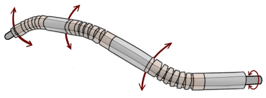 This work	★★★★☆ 1 DoF/seg × $n\ seg$	★★★★★ Motor base is 202 g	★★★★★ Multiple assembly configurations	★★★★★ decoupled BMs each BM fits constant curvature
DoF: Degree of freedom. Seg: Motion segment				

2. Details of the stainless-steel braided hose.

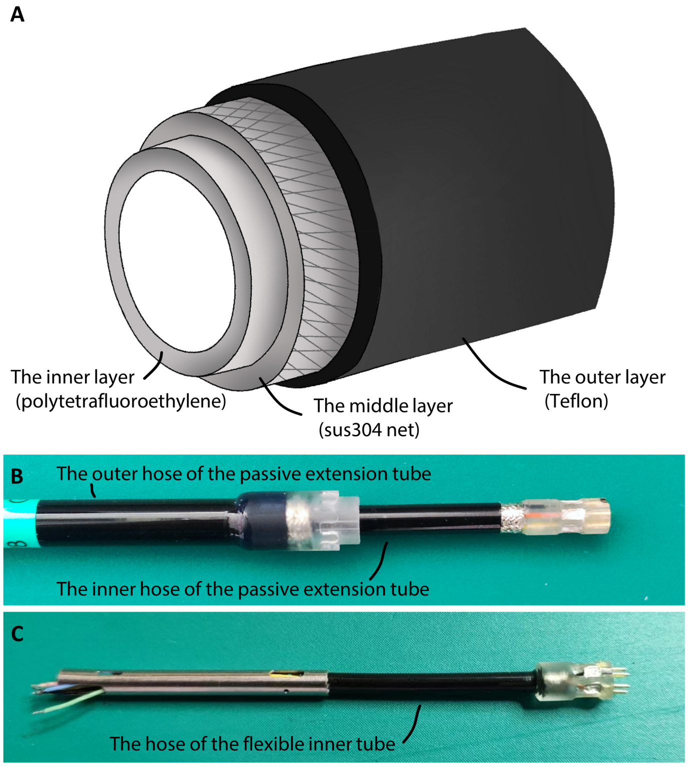

Supplementary Fig. 1. Details of the stainless-steel braided hose. (A) The illustration of the stainless-steel braided hose. **(B)** The passive extension tubes. **(C)** The flexible inner tube.

The stainless-steel braided hose contains three layers (Supplementary Fig. 1A). The inner layer provides lubrication for inserting other shafts. The middle layer is a braided SS304 net. This layer resists torque and prevents buckling. The inner layer and outer layer are made of Polytetrafluoroethylene (PTFE), which is characterized by cold and heat resistance (180-260°C), acid and alkali resistance, resistance to various organic solvents, and its friction coefficient is very low, making it suitable as a coating for endoscopes that come into direct contact with human tissue.

This kind of hose was used in the passive extension tube and adopted as the flexible section of the flexible inner tube. The main parameters are listed in Supplementary Table 2.

Supplementary Table 2. Parameters of the stainless-steel braided hoses

	Parameter	Values
The outer hose of the passive extension tube	Length	541 mm
	Outer diameter	6.1 mm
	Inner diameter	5.2 mm
The inner hose of the passive extension tube	Length	530 mm
	Outer diameter	3.9 mm

	Inner diameter	3.1 mm
The hose of the flexible inner tube	Length	33 mm
	Outer diameter	2.5 mm
	Inner diameter	1.7 mm

3. A schematic view of the planar steerable tube

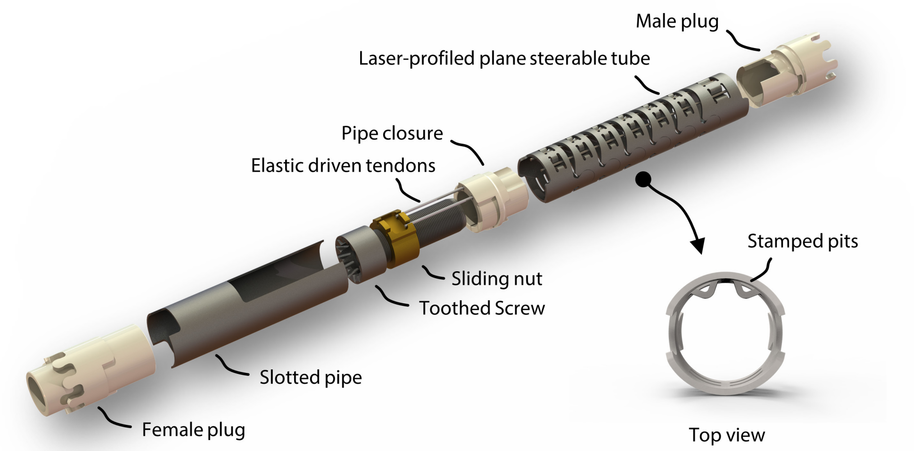

Supplementary Fig. 2. A schematic view of the planar steerable tube.

The planar steerable tube was divided into a rigid section and a steerable section. The steerable section was fabricated with laser-profile technology (LPT). The stamped pits to house the elastic drive tendons were presented in the top view of the pipe. The rigid section contained a slotted pipe. The sliding nut was actuated by the toothed screw and moved along the slot. Two ends of the planar steerable tube were closed with a female plug and a male plug separately. The two plugs had eight teeth. The connection of the plugs had eight phases.

4. The manufacturing process of the flexible inner tube.

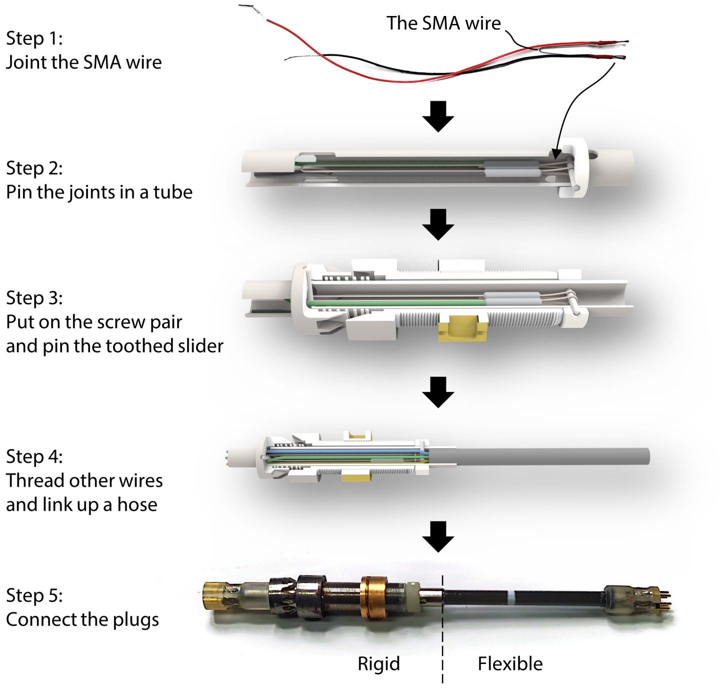

Supplementary Fig. 3. The manufacturing process of the flexible inner tube.

In the first step, two ends of the SMA wire were U-shaped and tied by two electric wires. The joints of the SMA wire were covered by insulation varnish. Secondly, the U-shape joints were pinned in a rigid tube. The SMA wire was bent to a U-shape. Thirdly, the screw pair, a compression spring, and a toothed slider were put on the rigid tube. The toothed slider was pinned. The pin traversed the slot on the tube and was hooked by the SMA wire. In the fourth step, a section of the stainless-steel braided hose was connected to the rigid tube. Six more wires were threaded through the whole tube. At last, a female plug sealed the left end of the rigid tube. A male plug sealed the right of the flexible hose. Each plug contained six pins. The wires were connected to the pins separately by the silver paste conductive adhesive.

The plugs of the planar steerable tube and the flexible inner tube were made of rigid resin by 3D-printing technology. Related parameters are listed in Supplementary Table 3.

Supplementary Table 3. Parameters of the rigid resin and 3D-print process

	Parameter	Values
Rigid resin	Hardness (D)	82
	Tensile Strength (MPa)	38
	Ultimate Elongation (%)	8
	Tensile Modulus (MPa)	1149

	Bending Strength (MPa)	49
	Flexural Modulus (MPa)	1476
Printing parameters	Nominal Resolution (μm)	35
	Slice Thickness (mm)	0.03
	Exposure time per layer (s)	1.2

5. The fabrication process of one bending module

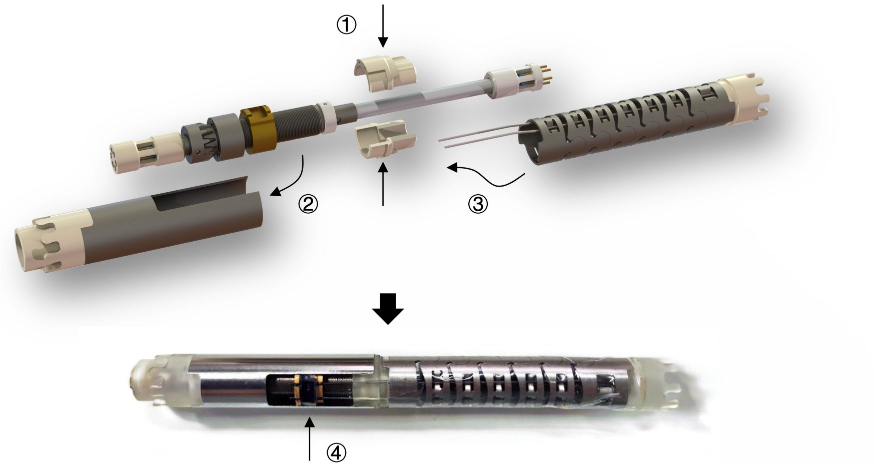

Supplementary Fig. 4. The fabrication process of one bending module.

The planar steerable tube was divided into a rigid section (left) and a steerable section (right). The flexible inner tube was embraced by two half-shells to constrain the axial movement. Then the flexible inner tube was inserted into the rigid section of the planar steerable tube and sealed by the half-shells. The steerable section was adhered to the half-shells from the right side. The driven tendons were threaded through two holes on the upper half-shell and stuck on the sliding nut.

6. A schematic view of the time-share driven robot

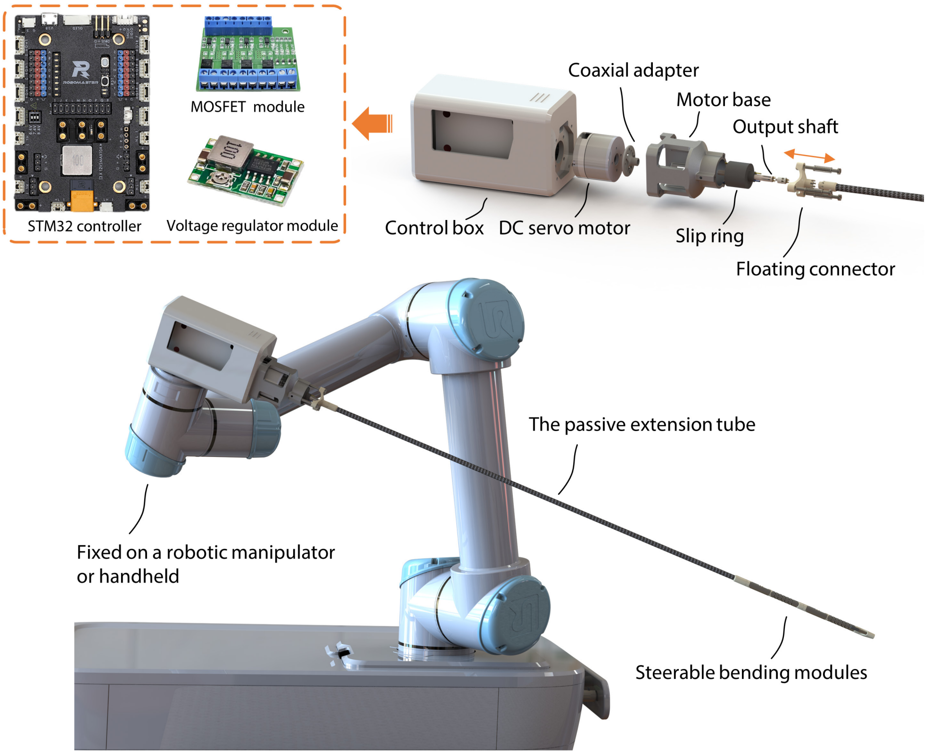

Supplementary Fig. 5. A schematic view of the time-share driven robot.

The time-share driven robot can be held by the operator or fixed on a robotic manipulator. The total weight of the control box and the motor base was 480 g. Inside the control box were the STM32 controller board, the Voltage regulator module, and the multi-channel MOSFET module with multi-channel PWM input from the STM32 controller. The motor base was attached to front side of the control box. The passive extension tube was attached to the motor base with the inner hose connected to the Output shaft. The outer hose was plugged in the Floating connector. The Floating connector contained three compression springs to adapt the relative length change of the outer hose with respect to the inner hose.

7. Modeling of one bending module.

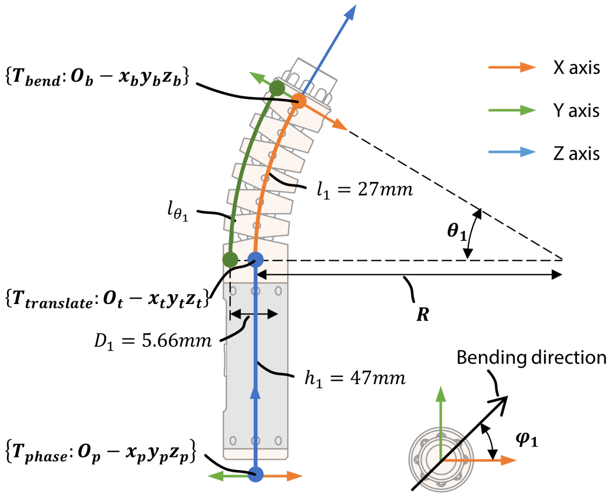

Supplementary Fig. 6. Modeling of one bending module.

The kinematic model of one bending module includes three transfer matrices.

The first is a rotation according to the installation phase of this segment

$$T_{phase} = \begin{bmatrix} cos\varphi_1 & -sin\varphi_1 & 0 & 0 \\ sin\varphi_1 & cos\varphi_1 & 0 & 0 \\ 0 & 0 & 1 & 0 \\ 0 & 0 & 0 & 1 \end{bmatrix} \tag{S1}$$

The second is a translation along the straight outer tube

$$T_{translate} = \begin{bmatrix} 1 & 0 & 0 & 0 \\ 0 & 1 & 0 & 0 \\ 0 & 0 & 1 & h_1 \\ 0 & 0 & 0 & 1 \end{bmatrix} \tag{S2}$$

where the h_1 represents the length of the rigid part.

The third is the bending motion

$$T_{bend} = \begin{bmatrix} \cos\theta_1 & 0 & \sin\theta_1 & l_1 \cdot (1 - \cos\theta_1)/\theta_1 \\ 0 & 1 & 0 & 0 \\ -\sin\theta_1 & 0 & \cos\theta_1 & l_1 \cdot \sin\theta_1 / \theta_1 \\ 0 & 0 & 0 & 1 \end{bmatrix} \tag{S3}$$

where, the l_1 represents the length of the bending part.

The transfer matrix of this bending module can be solved using the chain rule

$$T_{bm\,1} = T_{phase} \cdot T_{translate} \cdot T_{bend} \tag{S4}$$

$$
= \begin{bmatrix} cos\varphi_1 \cos\theta_1 & -sin\varphi_1 & cos\varphi_1 \sin\theta_1 & l_1 cos\varphi_1 \cdot \dfrac{(1-\cos\theta_1)}{\theta_1} \\ sin\varphi_1 \cos\theta_1 & cos\varphi_1 & sin\varphi_1 \sin\theta_1 & l_1 sin\varphi_1 \cdot \dfrac{(1-\cos\theta_1)}{\theta_1} \\ -\sin\theta_1 & 0 & \cos\theta_1 & l_1 \cdot \dfrac{\sin\theta_1}{\theta_1} + h_1 \\ 0 & 0 & 0 & 1 \end{bmatrix}
$$

where θ_1 is a function of l_{θ_1}, which represents the curve length of the driven cable in the bending part. In Supplementary Fig. 6, we have

$$R \cdot \theta_1 = l_1 \tag{S5}$$

and

$$(R + D_1/2) \cdot \theta_1 = l_{\theta_1} \tag{S6}$$

Minus Eq. S5 with Eq. S6, we got

$$\theta_1 = 2\left(l_{\theta_1} - l_1\right)/D_1 \tag{S7}$$

The kinematic model of one bending module can be represented by Eq. S4, and θ_1 can be determined by Eq. S7. Other constants are marked in Supplementary Fig. 6.

8. Modeling of the time-share driven robot

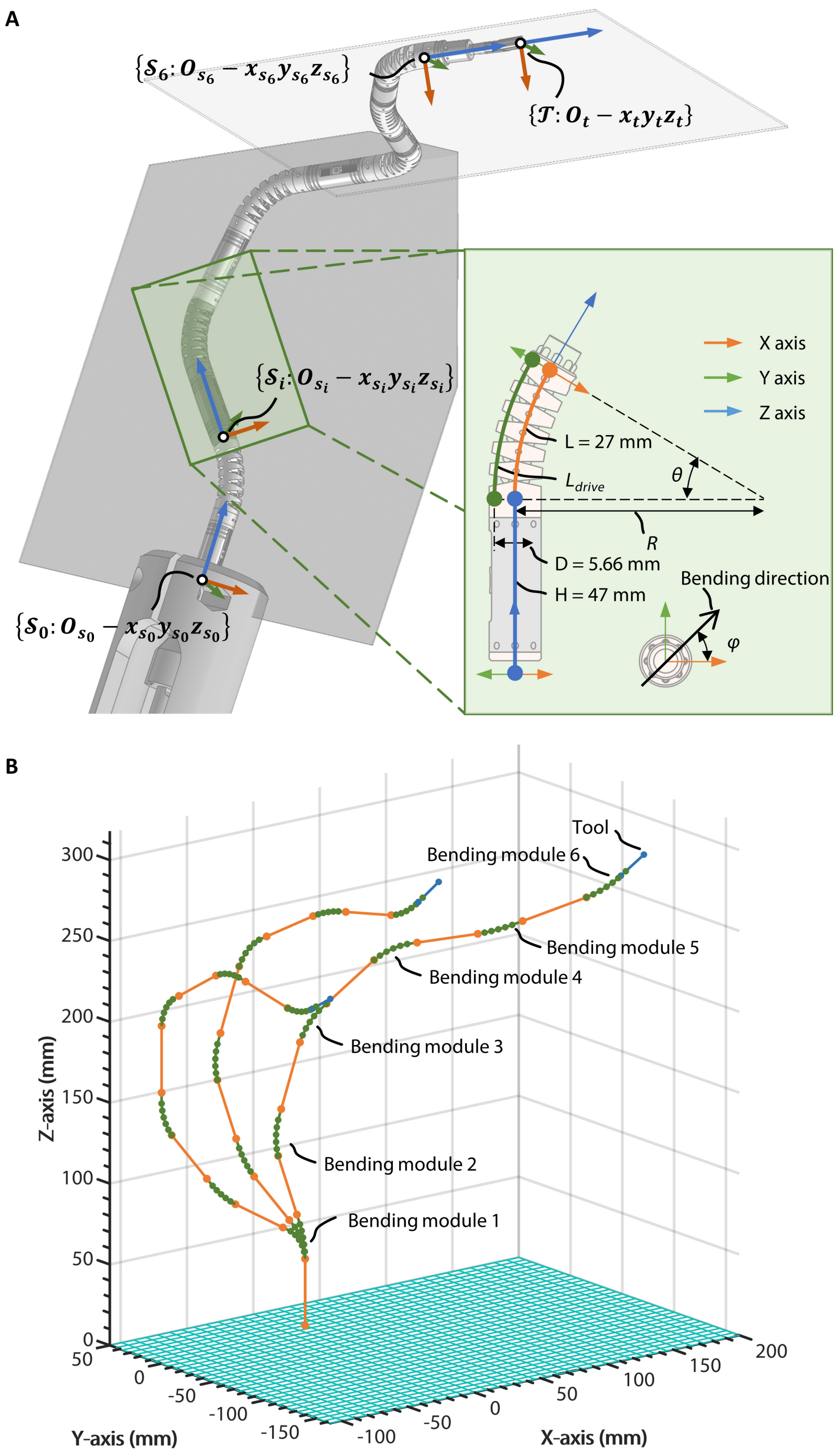

Supplementary Fig. 7. Modeling of the time-share driven robot. (**A**) The modeling of the time-share driven robot consisted of six BMs. (**B**) The kinematic model.

Considering the number of BMs and the assemble combination phases, the model of the time-share driven robot is determined by an installation vector $\boldsymbol{\varphi} = [\varphi_1 \quad \varphi_2 \quad \cdots \quad \varphi_n]$ and the design parameters of each BM. If not considering the

extension tube, according to Eq. S18, the tip position of the time-share driven robot can be represented as

$$T_{robot} = T_{bm\,1}(l_1, \quad h_1, \quad \varphi_1, \quad \theta_1) * \cdots * T_{bm\,n}(l_n, \quad h_n, \quad \varphi_n, \quad \theta_n) * T_{tool} \quad (S8)$$

Once the installation vector is settled, the tip position is only determined by $\boldsymbol{\theta}$

$$T_{robot} = T_{bm\,1\sim n}(\theta_1 \quad \theta_2 \quad \cdots \quad \theta_n) * T_{tool}$$
$$\theta_i = 2(p \cdot r_i - l_i)/D_i \quad (S9)$$

where, according to Eq. S9, p is the lead of the lead screw. Here, the lead is equal to the helical pitch (0.25 mm). r_i is the number of turns of each screw pair.

9. Demonstration of diagnosis experiment in a human stomach model

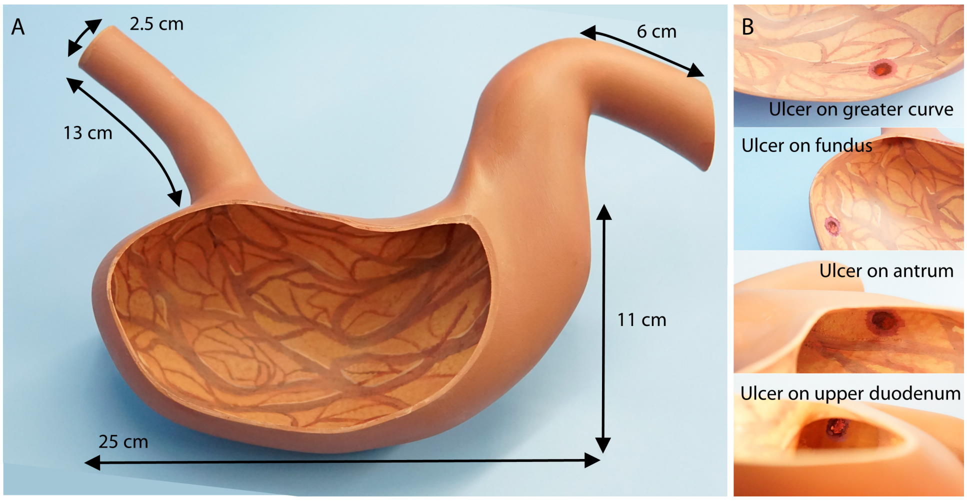

Supplementary Fig. 8. Demonstration of diagnosis experiment in a human stomach model.

(**A**) The human stomach model used in the diagnostic experiment was 3D printed. The stomach model was 25 cm in length and 11cm in width. The continuous esophagus was 13 cm long and the inner diameter was 2.5 cm. The continuous upper part of the duodenum was 6 cm. The outer surface of the model was painted to simulate the stomach, vessels and mucosa were drawn on the inner surface of the model to simulate the morphology of gastric mucosa. These were helpful to tip positioning or guide the motion direction in endoscopic view by the operator. (**B**) Ulcers were drawn on the inner wall of the stomach model at four anatomical locations as lesions in diagnosis experiments.

10.Demonstration of therapy experiment in an excised porcine stomach

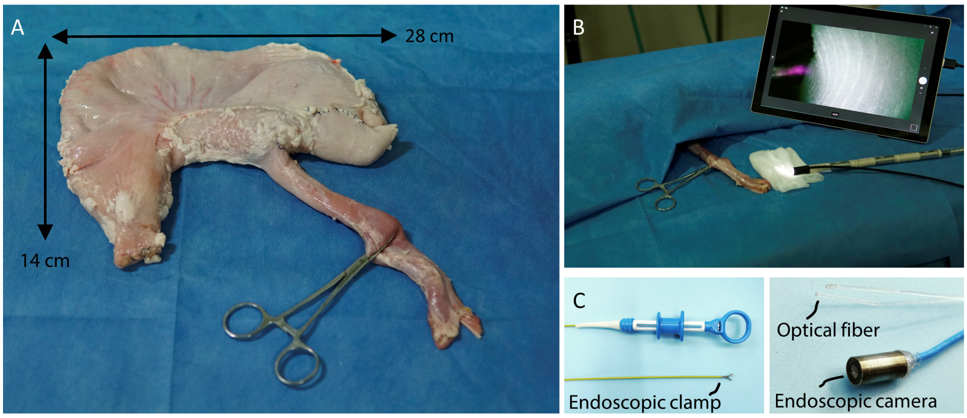

Supplementary Fig. 9. Demonstration of therapy experiment in an excised porcine stomach.

(**A**) The excised porcine stomach used in the therapy experiment was obtained from the Laboratory Animal Center of Zhejiang University with a length of 28 cm and a width of 14 cm. The diagnosis and therapy experiments were performed by one clinician with extensive experience in gastroscopy in the Microanatomy Laboratory of the Second Affiliated Hospital of Zhejiang University School of Medicine. There was a tear in the fundus of the porcine stomach, which was sutured properly and left a small opening for an external camera. In addition, there was a small opening in the pylorus for the inflation tube. The pylorus was also closed by a suture preventing air leaks in the experiment. (**B**) The setup of elements in therapy experiment. (**C**) Endoscopic clamps from Micro-Tech (Nanjing) with a diameter of 2.0 mm. The diameter of the endoscopic camera is 4 mm.
