# Supplementary material for: Bioinspired handheld time-share driven robot with expandable DoFs
Source: Nat Commun. 2024 Jan 26;15:768. doi: 10.1038/s41467-024-44993-x (PMC10817928; doi:10.1038/s41467-024-44993-x)
Supplement: Supplementary file 3 — Description of Additional Supplementary Files [file 41467_2024_44993_MOESM3_ESM.pdf]

Description of Additional Supplementary Files

Legends of Supplementary Movies:

Supplementary Movie 1: The rotary transmission along the passive extension tube.

Supplementary Movie 2: The bending module unit steering and carrying weight.

Supplementary Movie 3: The SMA wire's contraction.

Supplementary Movie 4: The SMA clutch's working process.

Supplementary Movie 5: Temperature change of the SMA clutch.

Supplementary Movie 6: The assembling of the bending modules.

Supplementary Movie 7: The proposed robot marches through a tortuous path.

Supplementary Movie 8: The proposed robot operates a target on a remote site.

Supplementary Movie 9: Diagnosis experiment in human stomach model.

Supplementary Movie 10: Therapy experiment in ex vivo porcine stomach.
